# LiNH_2_-Based Nitridation Synthesis and Structure Analysis of GaN:ZnO Solid Solutions

**DOI:** 10.3390/molecules30051134

**Published:** 2025-03-01

**Authors:** Ummul Khairat, Kazuhiro Manseki, Akito Ogawa, Takashi Sugiura

**Affiliations:** Graduate School of Natural Science and Technology, Gifu University, Yanagido 1-1, Gifu 501-1193, Japan

**Keywords:** nitridation, GaN:ZnO solid solution, nanoparticles

## Abstract

The GaN:ZnO solid solution is a visible-light-absorbing material widely developed for photocatalytic applications. For the first time, we demonstrate that a molecular source of LiNH_2_ significantly enhances the synthesis of GaN:ZnO solid solutions by leveraging its high reactivity in molten lithium chloride. Most notably, LiNH_2_ dramatically accelerates the nitridation reaction of gallium chloride (GaCl_3_) and zinc chloride (ZnCl_2_) or zinc oxide (ZnO), enabling the rapid formation of GaN:ZnO within just 2 h at a relatively low temperature of 650 °C. This marks a significant improvement over conventional ammonia gas synthesis methods, which typically require more than 10 hours. Furthermore, this approach eliminates the need for toxic ammonia gas and metal nitrate oxidizers, providing a safer and more environmentally sustainable pathway for material synthesis. Comprehensive structural and elemental analyses, including XRD, TEM, and XRF, confirmed the formation of highly crystalline GaN:ZnO solid solutions, revealing varying levels of reaction uniformity at the atomic scale under different zinc sources and Zn/Ga ratio conditions. The light absorption edges of the materials ranged from 500 nm to 650 nm, depending on the zinc content and source. These findings establish a novel and efficient synthesis strategy for GaN:ZnO solid solutions, paving the way for their development in visible-light-driven applications.

## 1. Introduction

Photocatalytic water splitting is considered a promising and sustainable approach for hydrogen fuel production, providing a pathway toward clean and renewable energy solutions [1,2]. To achieve efficient photocatalytic performance, numerous photocatalyst materials have been developed with a focus on meeting criteria, such as visible light absorption, suitable band-edge potentials to enable overall water splitting, and chemical stability under photocatalytic reaction conditions [3,4].

GaN:ZnO has emerged as one of the most promising materials for photocatalytic overall water splitting under visible light irradiation (λ > 400 nm) [5]. The material exhibits a narrower bandgap of approximately 2.3 eV, achieved through solid-state reactions, compared to the individual bandgaps of GaN (3.4 eV) and ZnO (3.0 eV) [6]. This unique optical property arises from the interaction between N 2p and Zn 3d orbitals, which elevates the valence band maximum of the solid solution [7]. Recent research has focused on the development of novel reaction systems and the fundamental investigation of their synthetic pathways using a solid-nitrogen source, as an alternative to the conventional NH_3_ gas-based processes. This conventional ammonia gas synthesis often takes more than 10 h. Moreover, it relies on toxic ammonia gas, which should ideally be replaced with a safer and more environmentally friendly process.

Previous studies have explored alternative pathways for synthesizing GaN:ZnO using solid-nitrogen sources, including combustion synthesis with urea (CO(NH_2_)_2_), which enables short reaction durations and allows for variation in the molar ratios of starting materials. However, this method requires metal nitrates (such as gallium nitrate and zinc nitrate) as oxidizers and often results in relatively large particle sizes on the micron scale [8]. Additionally, NH_4_Cl has been utilized as a nitrogen source to successfully shift the absorption edge of GaN:ZnO to the visible light region [9]. Another approach employed Zn_3_N_2_ in a two-step reaction to synthesize GaN/ZnO solid solutions, achieving visible light absorption [10]. Despite these advancements, most of the reported reaction times are prolonged (typically exceeding ten hours), highlighting the need for developing novel and efficient reaction systems.

While various methods incorporating solid nitrogen sources have demonstrated bandgap reduction in GaN:ZnO, a comprehensive understanding of the reaction mechanisms—particularly those involving solid nitrogen sources—is yet to be fully established. This study aims to elucidate the reaction process involved in forming a GaN/ZnO solid solution, particularly by utilizing lithium amide as a solid nitrogen source. In this study, we report a highly efficient synthesis of GaN:ZnO, achieving a remarkably short reaction time of only 2 h. This method not only extends light absorption into longer wavelengths compared to pristine GaN and ZnO but also provides systematic insights into the influence of different zinc sources on atomic-scale uniformity in the formation of GaN:ZnO.

## 2. Result and Discussion

### Synthesis and Structure Characterization of GaN:ZnO

**Figure 1:**
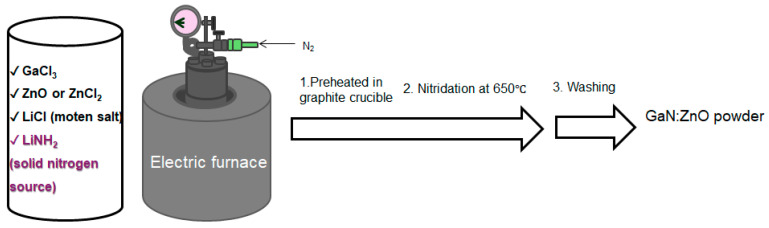
A reaction scheme for synthesizing GaN:ZnO solid solutions.

In our solid-state synthesis of GaN:ZnO, as illustrated in Figure 1, we identified LiNH_2_ as an effective molecular nitrogen source at relatively mild reaction temperatures as low as 650 °C. To optimize the nitridation conditions, we investigated two different zinc sources, ZnCl_2_ and ZnO, with Zn/Ga molar ratios of 0.5, 1, and 2, respectively (see Section 3 for details).

**Figure 2:**
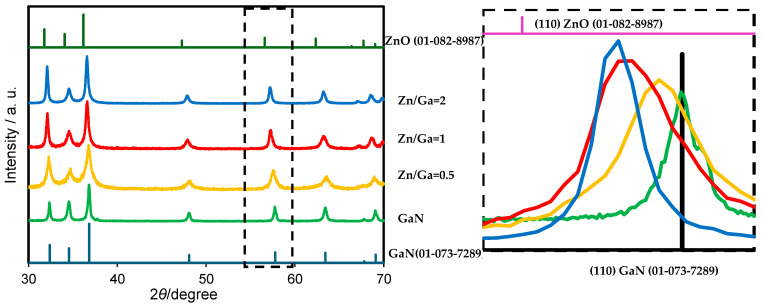
XRD pattern of the GaN/ZnO solid solution synthesized using ZnCl_2_ as the zinc precursor. The region of XRD peaks marked by the dotted square in the right figure corresponds to that in the left figure.

**Figure 3:**
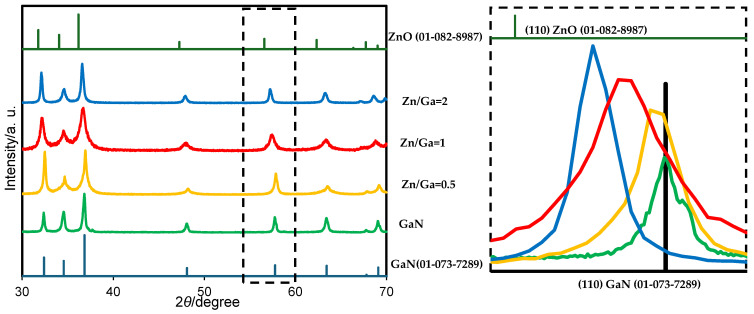
XRD pattern of the GaN/ZnO solid solution synthesized using ZnO as the zinc precursor. The region of XRD peaks marked by the dotted square in the right figure corresponds to that in the left figure.

Figure 2 and Figure 3 illustrate the crystalline phase of the GaN/ZnO solid solution. By varying the composition of gallium and zinc sources and selecting either zinc oxide or zinc chloride, the characteristic peaks of GaN and ZnO can be identified. The crystalline phase, representing a mixture of GaN and ZnO, is indicated by the position of the main peak, which lies between the peaks of the individual crystalline phases of GaN and ZnO. The (110) crystal plane of the product aligns between the reference patterns of wurtzite-type GaN (01-073-7289) and ZnO (01-082-8987). This suggests the successful formation of a solid solution, as confirmed by the main peak analysis in the XRD results. 

The use of molten lithium chloride provides a favorable chemical reaction environment due to its relatively low melting point [11], which enables it to melt under nitridation temperatures and enhance the reaction rate of the precursor materials. Consequently, lithium chloride plays a pivotal role in facilitating the formation of the GaN:ZnO solid solution. Furthermore, when using both zinc chloride and zinc oxide as starting materials, an increase in zinc content improves the crystallinity of the GaN:ZnO solid solution, as indicated by the enhanced intensity of the (110) diffraction peaks.

As shown in Table 1, samples with higher zinc content exhibited colors ranging from darker yellow to pale orange, in contrast to the Zn/Ga 0.5 samples. EDX analysis, as discussed later, indicates that the use of the zinc-rich precursor material, ZnO, led to the formation of a zinc-rich product.

**Figure 4:**
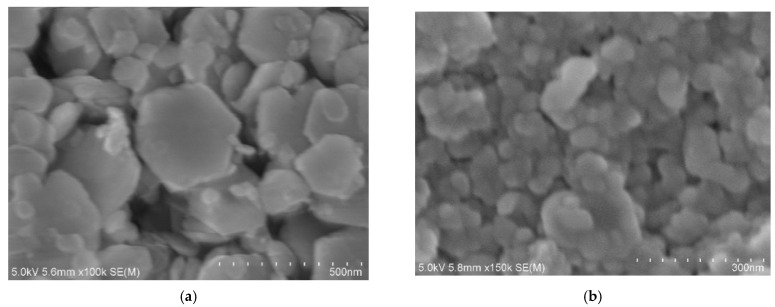

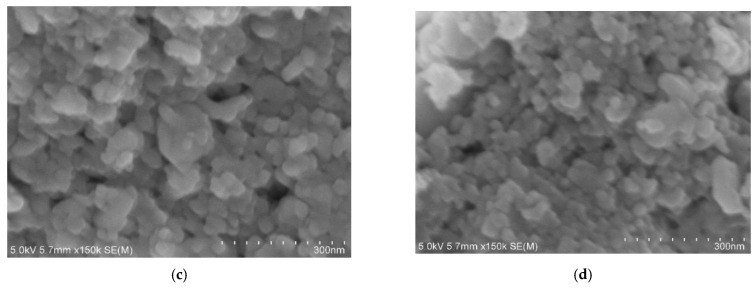
SEM images of GaN:ZnO samples synthesized under varying Zn/Ga ratios using ZnCl_2_ as the precursor: (**a**) GaN, (**b**) Zn/Ga = 0.5, (**c**) Zn/Ga = 1, and (**d**) Zn/Ga = 2.

SEM measurements clearly revealed a trend in the nitridation reactions, showing the formation of GaN:ZnO particles with varying sizes. Without the use of a zinc source, typical hexagonal-shaped particles characteristic of GaN were obtained, with the majority of the nanoparticles in the main products having dimensions of approximately 300 nm (Figure 4a). In contrast, when ZnCl_2_ was used as the zinc source, the grain sizes of the particles progressively decreased from around 100 nm to 50 nm as the zinc content increased (Figure 4b–d). This reduction in particle size may be attributed to the increased presence of chloride ions in the reaction, which potentially suppressed crystal growth.

**Figure 5:**
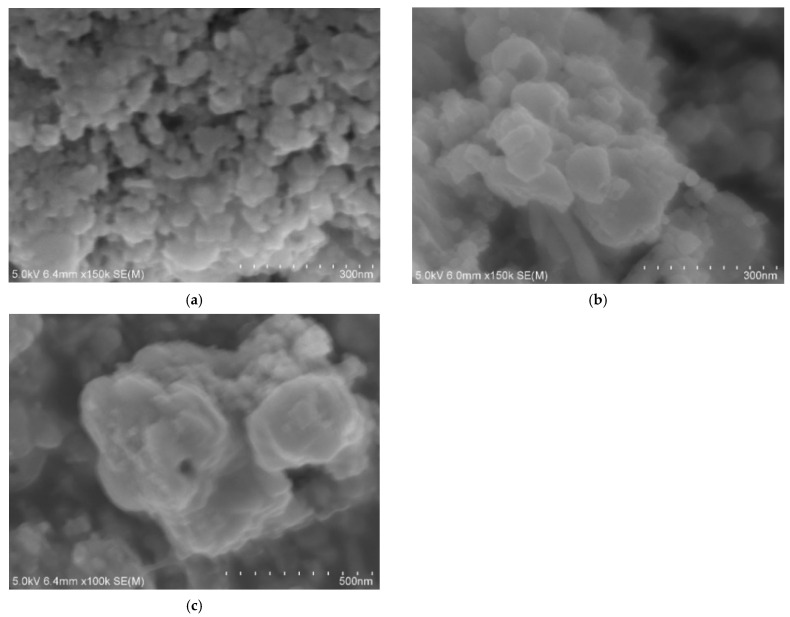
SEM images of GaN:ZnO samples synthesized with different Zn/Ga molar ratios using ZnO as the precursor: (**a**) Zn/Ga = 0.5, (**b**) Zn/Ga = 1, and (**c**) Zn/Ga = 2.

When ZnO was used as the zinc source, two distinct trends in particle size were observed under varying zinc content conditions, as shown in Figure 5a–c. A lower ZnO content resulted in smaller particles of around 30 nm, whereas higher ZnO content led to randomly shaped nanoparticles with a wide range of sizes, from tens of nanometers to the submicron scale. Notably, no hexagonal-shaped particles were observed in the solid solutions for either zinc source, indicating significant elemental diffusion during the formation of GaN:ZnO.

Based on Scherrer’s Equation (1), the crystallite sizes were determined, where D presents the crystallite size. K, λ, β, and θ show the Scherrer constant (0.90), X-ray wavelength (1.54 Å), FWHM of XRD peaks, and Bragg angle, respectively.

D = Kλ/βcosθ(1)

The crystallite sizes were estimated to range from 11 to 24 nm, as summarized in Table 2. It was observed that the crystallite sizes showed a decreasing trend with increasing zinc content under both zinc source conditions.

**Figure 6:**
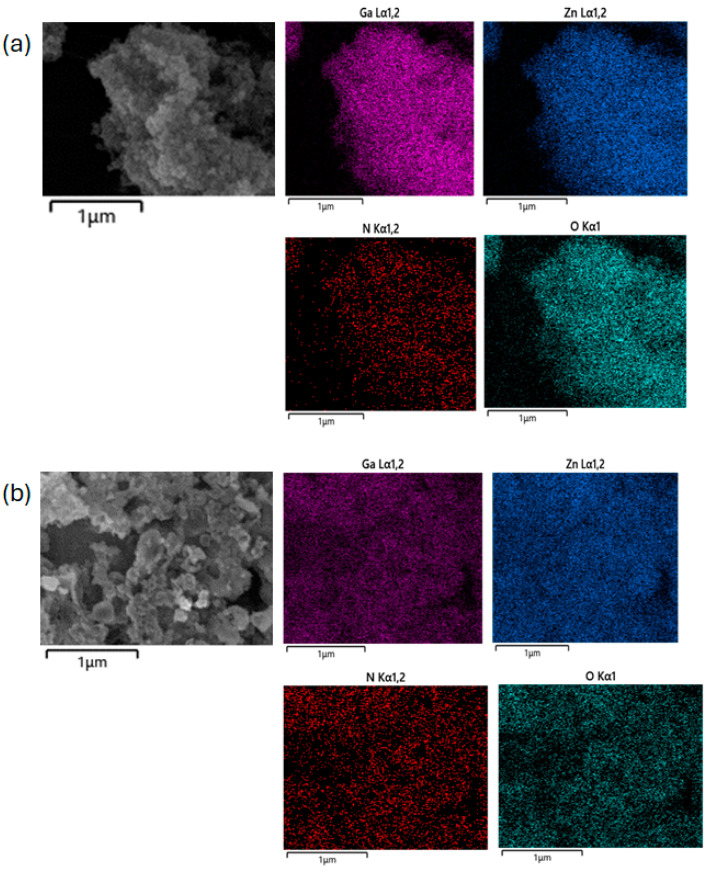
Energy Dispersive X-Ray Spectroscopy (EDS) analysis of GaN:ZnO solid solution samples prepared under Ga/Zn = 2 conditions. Zinc sources: (**a**) ZnCl_2_ and (**b**) ZnO.

Figure 6 presents the elemental mapping data for both zinc sources (Ga/Zn = 2), demonstrating the formation of a GaN:ZnO solid solution synthesized using the molecular solid-nitrogen source LiNH_2_.

**Figure 7:**
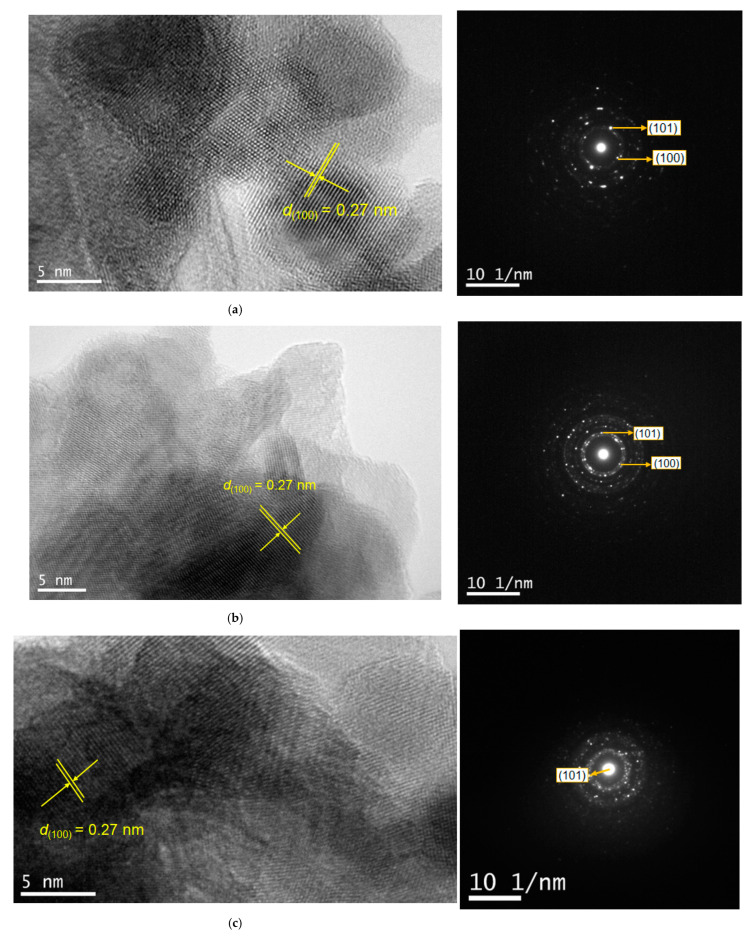

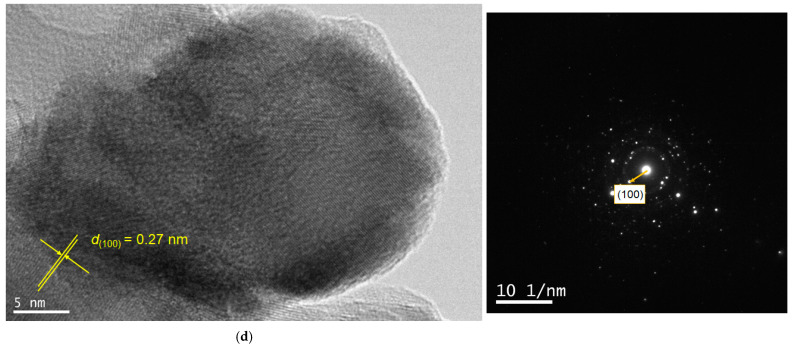
TEM images and SAD patterns of GaN:ZnO samples: (**a**) Zn/Ga = 0.5, zinc source: ZnCl_2_; (**b**) Zn/Ga = 0.5, zinc source: ZnO; (**c**) Zn/Ga = 2.0, zinc source: ZnCl_2_; and (**d**) Zn/Ga = 2.0, zinc source: ZnO.

To gain deeper insight into the LiNH_2_-based reactions, HR-TEM measurements were performed on samples with Zn/Ga ratios of 0.5 and 2 (Figure 7). For the Zn/Ga = 0.5 sample (Figure 7a,b), diffraction spots appeared prominently in specific rings of the selected area diffraction (SAD) pattern. Furthermore, lattice fringes corresponding to the (100) planes were identified in the TEM images, suggesting homogeneous diffusion of elements at the atomic scale.

Notably, the SAD patterns for higher zinc content samples (Figure 7c,d) indicated variations in the composition of Ga and Zn within the solid solutions. This observation is supported by the presence of more irregular diffraction spots along a ring corresponding to a specific crystal phase, suggesting compositional inhomogeneity at the atomic scale for Zn/Ga = 1 and 2 samples.

Additionally, XRF measurements were conducted (Table 3). The combined analysis of XRF and TEM data suggested that ZnCl_2_ provides a more efficient reaction environment for incorporating Zn at the atomic scale compared to ZnO in the formation of GaN/ZnO solid solutions. Regarding the Zn/Ga ratios, there is a tendency for lower Zn content after synthesis in all 6 samples compared to the synthesis conditions with different Zn/Ga ratios. It was observed that approximately 64%, 30%, and 58% of the expected Zn content from the starting ZnCl_2_ source reacted in the formation of the GaN:ZnO solid solution for Zn/Ga ratios of 0.5, 1, and 2, respectively. Additionally, 6%, 43%, and 23% of the starting ZnO source reacted for Zn/Ga ratios of 0.5, 1, and 2, respectively.

Considering the size discrepancies between the estimated crystallite sizes (11–24 nm) and the larger particles observed in SEM images (~50–100 nm for ZnCl_2_ and 30 nm to submicron scale for ZnO starting materials), it is likely that all synthesized particles are polycrystalline. Indeed, TEM images revealed that the primary nanoparticles had dimensions comparable to the estimated crystallite sizes and exhibited aggregation behavior.

In terms of crystal growth mechanisms, the nitridation process using LiNH_2_ without a zinc source proceeds according to the following Equations (2) and (3)), resulting in the formation of GaN, as reported in our previous study [12].

2GaCl_3_ + 6LiNH_2_ → 2Li_3_GaN_2_ + 3Cl_2_ + 6H_2_ + N_2_ (2)

2Li_3_GaN_2_ + 3Cl_2_ → 2GaN + 6LiCl + 2N_2_ (3)

In the case of the ZnCl_2_ source, a trace amount of oxygen in the reaction atmosphere likely leads to the formation of ZnO, which may occur concurrently with reaction (2), subsequently contributing to the production of GaN:ZnO via reaction (3). On the other hand, when ZnO is used as the zinc source, it can directly react with GaN through reaction (3) to form GaN:ZnO.

**Figure 8:**
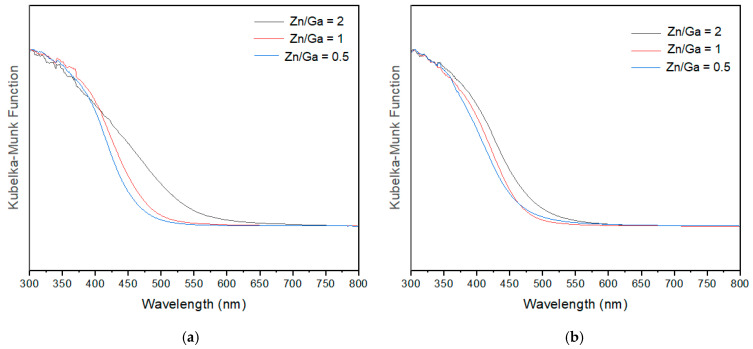
Diffuse-reflectance UV-Vis spectra of GaN:ZnO solid solution in 2 h nitridation time by using zinc sources: (**a**) ZnCl_2_ and (**b**) ZnO.

To investigate the optical properties of the obtained GaN/ZnO samples, absorption spectra were measured, as shown in Figure 8. The results revealed that longer-wavelength light, up to approximately 650 nm, can be absorbed for samples with a Zn/Ga ratio of 2, while samples with lower zinc content exhibited absorption at shorter wavelengths, around 500 nm. The results showed that using ZnCl_2_ enhances visible light absorption as the zinc content increases, in comparison to using ZnO as the source. The observed visible light absorption in Figure 8 is attributed to the incorporation of nitrogen into the crystal lattice, which shifts the valence band to a more negative energy level, thereby reducing the energy gap between the ground and excited states. According to the literature, this phenomenon can be explained by the interaction between N 2p and Zn 3d orbitals during the nitridation process [13,14,15].

Notably, this nitridation process, completed within just 2 h, emphasizes the critical role of LiNH_2_ in molten LiCl as the reaction medium. This study explores a novel synthesis approach that employs LiNH_2_ as a molecular solid-nitrogen source, enabling a significant reduction in reaction time compared to conventional ammonia-based synthesis methods, which often require more than 10 h [16,17] (Table 4). Furthermore, this work not only demonstrates an accelerated reaction but also expands the previously limited range of solid nitrogen sources, which had been restricted to urea [8], NH_4_Cl [9], and Zn_3_N_2_ [10]. Moreover, this approach eliminates the need for toxic ammonia gas and oxidizers such as metal nitrates in the urea-based reaction system [8], providing a safer and more environmentally friendly route for material synthesis. 

## 3. Materials and Methods

### 3.1. Chemicals

Gallium (III) chloride (GaCl_3_, 99.999%) was acquired from Yamanaka Hutech Corporation (Kyoto, Japan). Lithium Amide (LiNH_2_, 95%) was purchased from Sigma-Aldrich, Co., St. Louis, MO, USA. Zinc Chloride (ZnCl_2_) and Ethanol (99.5%) were supplied from Kanto Chemical Co., Inc., Tokyo, Japan. Lithium Chloride Anhydrous (LiCl) was sourced from Tokyo Chemical Industry Co., Ltd. in Tokyo, Japan. Zinc Oxide (ZnO) was derived from Nacalai Tesque, Inc., Kyoto, Japan. H_2_O (resistivity: 18.2 MΩ·cm) was obtained using a Milli-Q^®^ integral water purification system (MERK Ltd., Tokyo, Japan). Concentrated nitric acid (1.38) was procured FUJIFILM Wako Pure Chemical Corporation (Osaka, Japan). All chemicals were used without further purification.

### 3.2. Synthesis of GaN:ZnO Solid Solution Nanoparticles

The molar ratios of Zn/Ga were set to 0, 0.5, 1, and 2 for both zinc sources. For Zn/Ga = 0.5, 1.36 g of ZnCl_2_ and 3.52 g of GaCl_3_ were used, while for the zinc oxide source, 0.814 g of ZnO and 3.52 g of GaCl_3_ were employed. Similar calculations were applied for Zn/Ga ratios of 0, 1, and 2. The molar ratio of the other starting materials was maintained at 6:1 for LiNH_2_ (solid nitrogen source) and LiCl (molten salt source), corresponding to 1.38 g of LiNH_2_ and 0.423 g of LiCl, respectively.

All precursor materials were mixed through grinding in a glove box under a nitrogen atmosphere with a humidity level of 10%. The mixture was placed in a graphite crucible (inner diameter: 55 mm, length: 309 mm, SUS 316). A preheating process was conducted in an electric furnace (Ozawa Science Co., Ltd., Aichi, Japan) at 150 °C for 1 h under vacuum conditions. This was followed by a nitridation process at 650 °C for 2 h. After nitridation, the system was allowed to cool naturally to room temperature at a ramping rate of 10 °C/min.

To remove by-products, the yellow powder was washed sequentially with 1 M HNO_3_ and ethanol (EtOH) using a centrifuge operated at 4500 rpm for 15 min. The cleaned powder was then dried on a hotplate at 85 °C for approximately 30 min.

### 3.3. Characterization of GaN: ZnO Solid Solution Nanoparticles

X-ray diffraction (XRD) measurements were performed using a Rigaku RINT Ultima/PC apparatus with monochromated Cu–Kα radiation (Rigaku Corporation, Tokyo, Japan). The GaN:ZnO solid solution particles were characterized by scanning electron microscopy with energy-dispersive X-ray spectroscopy (SEM-EDX, S-4800, Hitachi High-Tech Corporation, Tokyo, Japan), X-ray fluorescence (XRF) (S8 Tiger, Blue Car AEX, Germany), and transmission electron microscopy (TEM, JEM-2100, JEOL Ltd., Tokyo, Japan). Diffuse reflectance spectra were recorded using a UV-Vis spectrophotometer (V-700, JASCO Corporation, Tokyo, Japan).

## 4. Conclusions

In conclusion, this study highlights the crucial role of the molecular solid-nitrogen source, LiNH_2_, in promoting the nitridation reaction at 650 °C. This approach enabled the rapid synthesis of GaN:ZnO solid solutions within just 2 h, yielding tunable light absorption edges ranging from 500 nm to 650 nm, depending on the zinc content and source. A comprehensive investigation of two different zinc sources in combination with LiNH_2_ provided valuable insights into the nitridation mechanisms governing GaN:ZnO formation, particularly the uniformity of reactions at the atomic scale. In addition, this method removes the necessity for toxic ammonia gas and oxidizing agents such as metal nitrates, offering a safer and more environmentally sustainable pathway for material synthesis. These findings deepen our understanding of the synthesis process and offer strategies for optimizing GaN:ZnO materials for diverse applications.

## Figures and Tables

**Table 1:** Photographs of GaN/ZnO solid solution powder samples synthesized using two different zinc precursor materials with varying Zn/Ga ratios.

	Zn/Ga	Zn/Ga = 0.5	Zn/Ga = 1	Zn/Ga = 2
Zn Sources				
ZnCl_2_		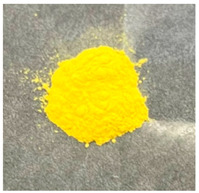	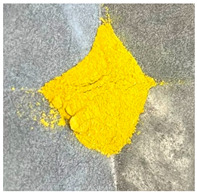	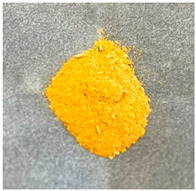
ZnO		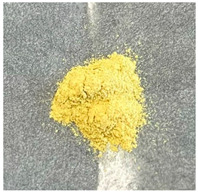	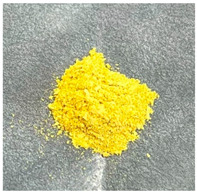	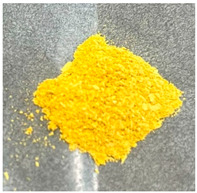

**Table 2:** Crystallite sizes of GaN:ZnO solid solutions synthesized using ZnCl_2_ and ZnO as zinc sources, estimated from XRD patterns.

Zn/Ga	Zn Source: ZnCl_2_	Zn Source: ZnO
0.5	23 nm	24 nm
1	19 nm	20 nm
2	11 nm	21 nm

**Table 3:** The Zn/Ga ratios of GaN:ZnO solid solutions determined by XRF measurements.

Zn/Ga	Zn Source: ZnCl_2_	Zn Source: ZnO
0.5	Ga_0.79_Zn_0.21_	Ga_0.98_Zn_0.02_
1	Ga_0.85_Zn_0.15_	Ga_0.79_Zn_0.21_
2	Ga_0.61_Zn_0.39_	Ga_0.85_Zn_0.15_

**Table 4:** Examples of various nitridation methods for synthesizing GaN:ZnO solid solutions.

Nitrogen Sources	Metal Ion Sources	Reaction Temperatures	Reaction Times	References
NH_3_ gas	Ga_2_O_3_ and ZnO	850 °C	5–30 h.	[16]
NH_3_ gas	Ga(NO_3_)_3_ and Zn(CH_3_CO_2_)_2_	650~850 °C	10 h.	[17]
Urea	Ga(NO_3_)_3_ and Zn(NO_3_)_2_	350~800 °C	30 min.	[8]
NH_4_Cl	Ga_2_O_3_ and Zn	600~850 °C	2–50 h.	[9]
Zn_3_N_2_	Ga_2_O_3_ and Zn_3_N_2_	650~1000 °C	10 h.	[10]
LiNH_2_	GaCl_3_ and ZnO or ZnCl_2_	650 °C	2h.	This work

## Data Availability

The original contributions presented in the study are included in the article, further inquiries can be directed to the corresponding author.

## References

[1] Jia G., Sun F., Zhou T., Wang Y., Cui X., Guo Z., Fan F., Yu J.C. (2024). Charge Redistribution of a Spatially Differentiated Ferroelectric Bi_4_Ti_3_O_12_ Single Crystal for Photocatalytic Overall Water Splitting. Nat. Commun..

[2] Wang Z., Lin Z., Shen S., Zhong W., Cao S. (2021). Advances in Designing Heterojunction Photocatalytic Materials. Chin. J. Catal..

[3] Fang Z., Chen G., Zhu G., Shan L., Xu H., Suriyaprakash J., Wu H., Dong L., Li X., Lu C. (2025). Crystal Facet/Interface Anchored Janus Activity of BiOBr in Driving Photocatalytic Water Splitting. Sep. Purif. Technol..

[4] Bao Y., Li C., Domen K., Zhang F. (2022). Strategies and Methods of Modulating Nitrogen-Incorporated Oxide Photocatalysts for Promoted Water Splitting. Acc. Mater. Res..

[5] Maeda K., Domen K. (2010). Solid Solution of GaN and ZnO as a Stable Photocatalyst for Overall Water Splitting under Visible Light. Chem. Mater..

[6] Maeda K., Teramura K., Domen K. (2008). Effect of Post-Calcination on Photocatalytic Activity of (Ga_1–X_Zn_x_)(N_1–X_O_x_) Solid Solution for Overall Water Splitting under Visible Light. J. Catal..

[7] Hirai T., Maeda K., Yoshida M., Kubota J., Ikeda S., Matsumura M., Domen K. (2007). Origin of Visible Light Absorption in GaN-Rich (Ga_1–X_Zn_x_)(N_1–X_O_x_) Photocatalysts. J. Phys. Chem. C.

[8] Kennedy A.E., Meekins B.H. (2018). Combustion Synthesis and Photoelectrochemical Characterization of Gallium Zinc Oxynitrides. J. Mater. Res..

[9] Liu K., Zhang B., Zhang J., Lin W., Wang J., Xu Y., Xiang Y., Hisatomi T., Domen K., Ma G. (2022). Synthesis of Narrow-Band-Gap GaN:ZnO Solid Solution for Photocatalytic Overall Water Splitting. ACS Catal..

[10] Iwasa N., Sandaiji H., Nandy S., Nakabayashi M., Takata T., Hisatomi T., Domen K. (2024). Long-Wavelength Photoresponsive Gallium Zinc Oxynitride for Efficient Oxygen Evolution and Z-Scheme Water Splitting Reactions. J. Mater. Chem. A Mater..

[11] Purwiandono G., Manseki K., Sugiura T. (2020). A Molten Salt-Based Nitridation Approach for Synthesizing Nanostructured InN Electrode Materials. RSC Adv..

[12] Purwiandono G., Manseki K., Sugiura T. (2020). Photo-Electrochemical Property of 2D Hexagonal-Shape GaN Nanoplates Synthesized Using Solid Nitrogen Source in Molten Salt. J. Photochem. Photobiol. A Chem..

[13] Liu J., Fernández-Serra M.V., Allen P.B. (2016). Special Quasiordered Structures: Role of Short-Range Order in the Semiconductor Alloy (GaN)1-x(ZnO)x. Phys. Rev. B.

[14] Yuan X., Wu M., Ni J., Cheng Y., Ni C. (2023). Tuning the ZnO/GaN Heterojunction for Atmospheric NO Abatement. Appl. Surf. Sci..

[15] Huda M., Yan Y., Wei S.-H., Al-Jassim M. (2008). Electronic Structure of ZnO:GaN Compounds: Asymmetric Bandgap Engineering. Phys. Rev. B.

[16] Maeda K., Takata T., Hara M., Saito N., Inoue Y., Kobayashi H., Domen K. (2005). GaN:ZnO Solid Solution as a Photocatalyst for Visible-Light-Driven Overall Water Splitting. J. Am. Chem. Soc..

[17] Han W.Q., Liu Z., Yu H.G. (2010). Synthesis and Optical Properties of GaN/ZnO Solid Solution Nanocrystals. Appl. Phys. Lett..

